# Methylmercury, Amalgams, and Children’s Health

**DOI:** 10.1289/ehp.114-1392265

**Published:** 2006-03

**Authors:** Gianpaolo Guzzi, Claudio Minoia, Paolo D. Pigatto, Gianluca Severi

**Affiliations:** Italian Association for Metals and Biocompatibility Research, A.I.R.M.E.B., Milan, Italy, E-mail: gianpaolo_guzzi@fastwebnet.it; Laboratory of Environmental and Toxicology Testing “S. Maugeri”, IRCCS, Pavia, Italy; Department of Dermatological Sciences, University of Milan, IRCCS Maggiore Hospital, Foundation Policlinico, Milan, Italy; Cancer Epidemiology Centre, The Cancer Council Victoria, Melbourne, Australia

In their excellent article, Björnberg et al. (2005) stated that exposure to methyl-mercury in humans occurs primarily through fish consumption. We would like to make one observation about the sources of potential exposure to methylmercury in the general population.

We were surprised that Björnberg et al. (2005) failed to mention saliva as a plausible biologic source of methylmercury in individuals who have mercury dental fillings. Leistevuo et al. (2001) found a correlation between the total amalgam surfaces and organic mercury—presumably as methylmercury (CH_3_Hg^+^)—in saliva.

Previous studies have reported that mouth air levels of elemental mercury (Hg^0^) significantly correlate with the number of occlusal surfaces (Lorscheider et al. 1995; Clarkson 2002). Hence, when mercury vapor (Hg^0^) is released from amalgams and dissolved into the saliva, it exists mainly as Hg^0^ and partly as inorganic divalent mercury (Hg^2+^).

Consistent with this background, saliva has high levels of inorganic mercury associated with the total number of amalgam surfaces, which markedly increased during mastication and bruxism. In approximately 270 individuals with amalgams, we used inductively coupled plasma-mass spectrometry to measure a wide range of possible values of total mercury in saliva. Mercury levels ranged from the limit of detection [LOD; 0.1 μg/L] to 780 μg/L in both salivary baseline flow rate in unstimulated condition and in a post–chewing-gum test (Guzzi et al. 2005).

Trace amounts of elemental and inorganic mercury from saliva are taken up by oral bacteria, which in turn release methyl-mercury as their by-product. Heintze et al. (1983) and Lyttle et al. (1993) reported direct evidence that organic mercury in saliva is due to the transformation of bacteria. As shown in our article (Pigatto et al. 2005), the proximate cause of mercury alkylation in oral microbial communities—which occurs in dental plaque—appears to be associated with the presence of some bacteria.

Furthermore, our ongoing investigation seems to support the work of Leistevuo et al. (2001), suggesting evidence that subjects with dental amalgams have shown higher levels of methylmercury compared with controls (Guzzi et al. 2005).

Once ingested in the gastrointestinal tract, the methylmercury in saliva is therefore nearly all absorbed (> 95%), as is methylmercury in fish. Leistevuo et al. (2001) reported that the levels of methyl-mercury in saliva ranged from 0 to 174 nmol/L (0–37.523–μg/L), with a mean methylmercury level estimate of 14.0 nmol/L (3.019–μg/L). (Leistevuo et al. 2001). Assuming that daily adult salivary secretion is at least 800 mL, speciation analyses indicate that exposure to methyl-mercury through ingestion—apparently derived from oral bacteria biomethylation of inorganic mercury—is about 2–3 μg/day (Leistevuo et al. 2001). Perhaps Björnberg et al. (2005) did not deem this exposure significant?

Considering that the relevant feature of methylmercury in humans is accumulation in both adult and fetal brain, it is quite clear that, over time, the extensive exposure to methylmercury associated with dental amalgams should be taken into account. We believe that organic mercury found in saliva may indeed represent a potential nondietary source of methylmercury.
